# Changes in Frequency and Activation Status of Major CD4^+^ T-Cell Subsets after Initiation of Immunosuppressive Therapy in a Patient with New Diagnosis Childhood-Onset Systemic Lupus Erythematosus

**DOI:** 10.3389/fped.2017.00104

**Published:** 2017-05-15

**Authors:** Saimun Singla, Scott E. Wenderfer, Eyal Muscal, Anna Carmela P. Sagcal-Gironella, Jordan S. Orange, George Makedonas

**Affiliations:** ^1^Department of Pediatrics, Baylor College of Medicine, Houston, TX, USA; ^2^Division of Allergy, Immunology and Rheumatology, Texas Children’s Hospital, Houston, TX, USA; ^3^Renal Section, Texas Children’s Hospital, Houston, TX, USA

**Keywords:** T regulatory cells, T effector cells, childhood-onset systemic lupus erythematosus, T-cell exhaustion, PD-1

## Abstract

**Background:**

Several studies suggest that defects of regulatory T-cells (Tregs) and impaired cellular immunity are secondary to an imbalance between auto-aggressive T-cells and Tregs in lupus patients. Discrepancies in Tregs and effector T-cells (Teff) in active lupus patients are shown to be restored in patients upon receiving immunosuppressive therapy. Therefore, our main aim was to observe frequencies of these CD4^+^ T-cell subsets and Tregs/Teff ratio in a new diagnosis of childhood-onset systemic lupus erythematous (cSLE) before and after initiation of therapy. In addition, we monitored T-cell exhaustion status by examining responses to super-antigen staphylococcal enterotoxin B (SEB) and PD-1 expression in this patient.

**Methods:**

Phenotyping of CD4^+^ T-cell subsets was carried out under basal conditions and after SEB stimulation using flow cytometry in one inactive (I-cSLE) and one active cSLE (A-cSLE) patient, as well as a healthy control (HC). The A-cSLE patient was a new diagnosis. Variables were measured at three consecutive time points in the active patient, reflecting various stages of disease activity. Activation status of CD4^+^ T-cells in the A-cSLE patient was compared to that of the I-cSLE patient and HC. Disease activity was measured by calculating the systemic lupus erythematous disease activity index.

**Results:**

We found that the A-cSLE patient was not Tregs deficient. The patient had increased frequency of Tregs, and the Tregs/Teff ratio increased when the disease activity became less severe. CD4^+^ T-cells in the I-cSLE patient and in the A-cSLE patient with milder disease activity had heightened responsiveness to SEB, whereas T-cells were relatively hypo-responsive to SEB in the A-cSLE patient when disease activity was higher. The active patient exhibited higher frequencies of PD-1^+^ expressing Tregs, Teff, and Tnaïve/mem cells under basal conditions compared to the HC and I-cSLE patient.

**Conclusion:**

In the A-cSLE patient, changes in Tregs/Teff ratio correlated better with clinical improvement compared to Tregs frequencies alone and might reflect the restoration of immune homeostasis with therapy. SEB hypo-responsiveness in the A-cSLE patient when disease activity was higher paralleled with findings of greater frequencies of PD-1^+^ expressing Tregs, Teff, and Tnaïve/mem cells, suggests a possible global exhaustion status of CD4^+^ T-cells in this patient.

## Introduction

Systemic lupus erythematosus (SLE) is an autoimmune disease that includes both genetic and environmental components leading to an irreversible break in immune tolerance and attack against endogenous nuclear antigens in the genetically susceptible host (1–3). Literature suggests that failure in peripheral tolerance mechanisms may initiate this pathogenic process (4–6).

Adult studies demonstrate that the failure of peripheral tolerance can be due to various aberrancies, such as hyper-excitable T-cell receptors (TCR), altered inhibitory receptor expression patterns, and reduced numbers and/or impaired function of regulatory T-cells (Tregs) compared to healthy controls (HCs) (7–10). In contrast, Tregs have also been shown to be significantly expanded in patients with active SLE compared to those with inactive disease and HCs (10–13). Some studies suggest that the defects of Tregs are secondary to the imbalance between auto-aggressive T-cells and Tregs in lupus patients, tipping the balance toward Teffector (Teff) activation, clonal expansion, cell survival, cytokine production, and ultimately, autoimmunity (8, 11, 12).

Similarly, impaired cellular immunity in human SLE has also been noted as these lymphocytes can be non-responsive to mitogenic or antigenic stimuli *in vitro* compared to HCs (13). This defective cellular function is attributed to disease severity/duration, intrinsic T-cell defects, and exhaustion of previously activated cells (13). Interestingly, this expected exhaustion status becomes evident as these cells have reduced proliferation responses to TCR V β-specific super-antigen staphylococcal enterotoxin B (SEB) in chronic autoimmunity or infections (14). Some studies demonstrate that the T-cell exhaustion status is associated with higher expression levels of PD-1 (CD 279), a co-inhibitory molecule that negative regulates T-cell responses (15, 16).

Most studies describing aberrancies in SLE pathogenesis are adult driven, whereas similar data are relatively scarce for pediatric lupus patients, a cohort with higher mortality rates than their adult counterpart (17). Furthermore, methodology in most lupus studies involves comparing active versus inactive SLE patients at one fixed time point. This precludes a better appreciation of the longitudinal changes in the immune compartment during different stages of lupus disease activity.

In this case series, we report the longitudinal changes in CD4^+^ T-cell subsets Teff and Tregs using flow cytometry in a newly diagnosed childhood-onset systemic lupus erythematous (cSLE) patient at three consecutive time points that correlate with different stages of disease activity based on their SLE disease activity index (SLEDAI). We also report PD-1 expression on these subsets as well as T-cell responsiveness to SEB. The overall methodology of the study is described below followed by the results which include the clinical presentation of the active and inactive cSLE patients and the flow cytometric analyses of their CD4^+^ T-cell subsets.

## Materials and Methods

### Study Subjects

The active and inactive cSLE patients as well as the HC were recruited under an immune profiling research protocol approved by the Institutional Review Board of Baylor College of Medicine (#H33095). Participants provided written consent prior to sample collection. Peripheral blood mononuclear cells (PBMCs) were collected from the active cSLE (A-cSLE) patient at three consecutive time points and the inactive cSLE patient and HC at one time point. The A-cSLE patient was new diagnosis prior to any therapeutic intervention.

### PBMC Collection

Blood was collected from patients in ACD-containing tubes. PBMCs were isolated immediately after blood draws by Ficoll gradients (Ficoll-Paque PLUS; GE Healthcare) and cryopreserved in 90% FCS + 10% dimethylsulfoxide until use.

### PBMC Stimulation

Peripheral blood mononuclear cells were thawed, allowed to rest for 1 h in complete media (1 × 10^6^ cells/mL), and then stimulated with 0.5 µg/mL of super-antigen SEB (Millipore Corp.) for 24 h at 37°C in a CO_2_ incubator.

### Staining and Flow Cytometry Phenotypic Analysis

Both stimulated and unstimulated PBMCs were stained with AF700-conjugated anti-CD4 (eBioscience), BV 785-conjugated anti-CD8 (Biolegend), PECy5-conjugated anti-CD25 (BD Pharmingen), APC-Cy7-conjugated anti-PD-1 (Tonbo), and Ghost Dye™ Violet 510 (Tonbo) to stain for live cells. Cells were then treated with Foxp3 Fixation/Permeabilization Concentrate and Diluent (Tonbo) and stained with BV711-conjugated anti-CD3 (BD Horizon) and AF488-conjugated anti-Foxp3 (BD Pharmingen).

**Figure 1:**
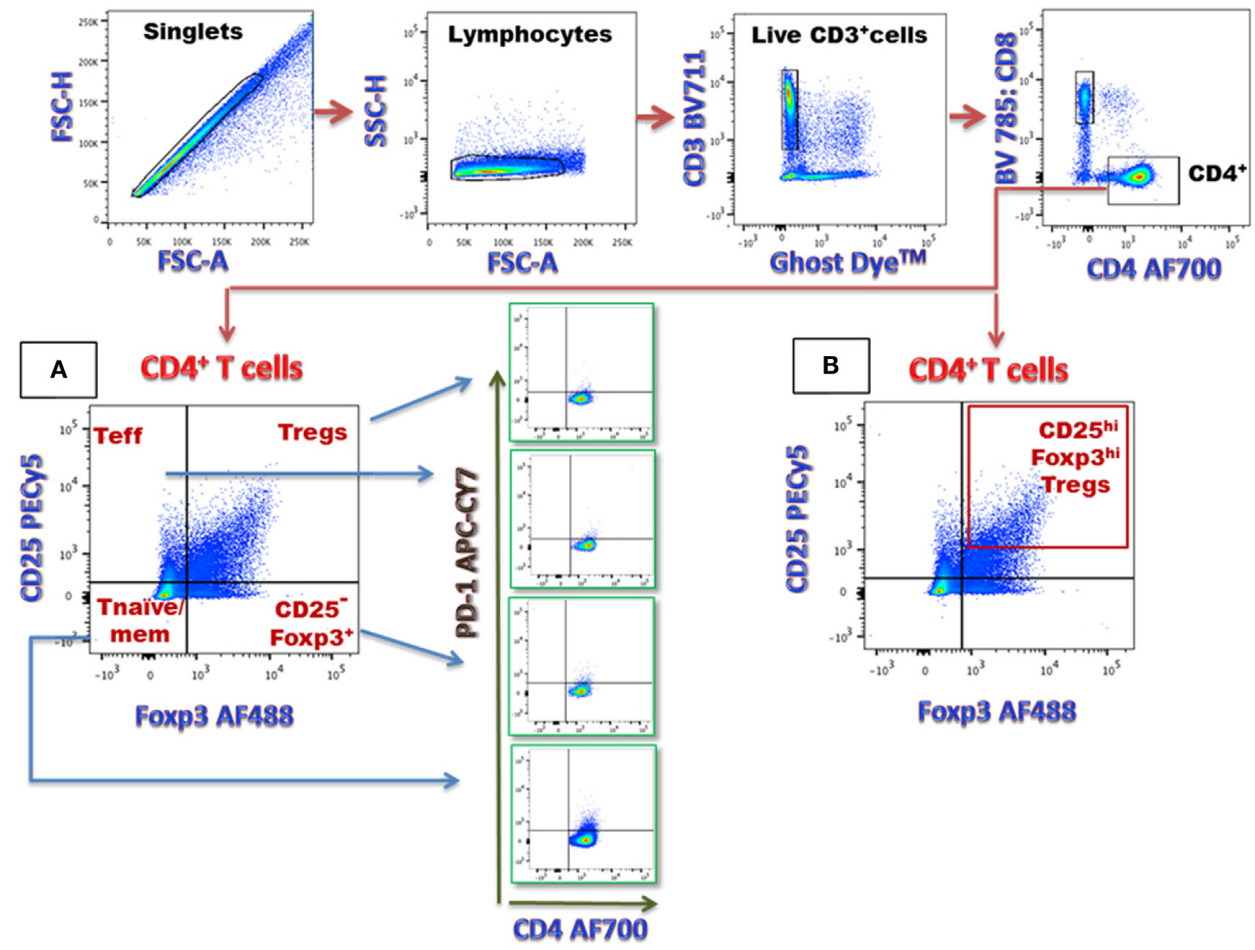
**Flow cytometric analysis of CD4^+^ T-cell subsets (A) in this sample gating, cells were first gated on singlets (FSC-H versus FSC-A) and lymphocytes (SSC-H versus FSC-A)**. The lymphocyte gate is further analyzed for live versus dead cells. The live lymphocytes (CD3^+^) were analyzed to identify CD4^+^ T-cell population (CD8^-^CD4^+^). This population was then analyzed for various T-cell subsets, including CD25^+^Foxp3^+^ [regulatory T-cells (Tregs)], CD25^+^Foxp3^−^ Teffector, CD25^−^Foxp3^−^ (T naïve/mem), and CD25^−^Foxp3^+^ (CD25^−^ Foxp3^+^) T-cells. PD-1 expressing CD4^+^ T-cell numbers were determined within each CD4^+^ T-cell subset. **(B)** Frequency of CD25^hi^ Foxp3^hi^ Tregs within the total Tregs pool is represented in the box in right upper quadrant.

After staining, fluorescence data were acquired *via* a BD LSRFortessa™ and analyzed *via* FlowJo software (v10.0.8, Tree Star Inc.) to assess for CD4^+^ T-cell subsets in the basal state and in response to SEB. General gating strategy for flow cytometry analysis is depicted in Figure 1A. Sub-analysis gating of CD4^+^CD25^hi^Foxp3^hi^ cells on Tregs is shown in Figure 1B.

### Disease Activity Score

Disease activity of patients from each time point was calculated using the SLEDAI (18–20). Scores greater than or equal to 6 were considered active disease.

### Approach to Phenotypic Analysis

We examined the pattern of frequencies of Teffector and Tregs, as well as the balance between these T-cell subsets at three consecutive time points that correlate with different stages of disease activity in the newly diagnosed cSLE patient. Time point 1 for this patient reflects results of a therapy-naïve immune system, whereas time points 2 and 3 are results obtained in the midst of immunosuppressive therapy (Table 1). Teff cells are defined as CD4^+^ T-cells with an activated phenotype (CD4^+^CD25^+^Foxp3^−^), naïve/mem are CD4^+^CD25^−^Foxp3^−^ and Tregs are CD4^+^ T regulatory cells (CD4^+^CD25^+^ Foxp3^+^). We also evaluated the frequency of CD4^+^CD25^hi^Foxp3^hi^ Tregs as they have been shown to exhibit more suppressive ability. This selection strategy also excluded a substantial proportion of naïve or resting Tregs that express lower levels of CD25 (21, 22).

**Table 1:** **Demographics and clinical characteristics of active and inactive childhood-onset systemic lupus erythematous (cSLE) patients**.

Patient	Gender/ethnicity	Age (years)	Time point	ACR classification criteria at initial cSLE diagnosis	SLEDAI at time of sample collection (normal ≤ 6)	Medications
Active cSLE patient	F/AA	9	1: day 1	AR, MUC, MR, HE, IM, ANA	14	N/A
			2: day 28		10	ASA,^a^ HCQ,^a^ IVP,^a^ MTX,^a^ PRED^a^
			3: day 80		8	ASA,^a^ HCQ,^a^ IVP, MTX,^a^ PRED^a^
Inactive cSLE patient	F/AA	17	1	AR, MR, MUC, LN, HE, IM, ANA	0	ASA,^a^ CYC, HCQ,^a^ IVP, MMF,^a^ PRED

*AA, African-American; ACR, American College of Rheumatology; SLEDAI, Systemic Lupus Erythematosus Disease Activity Index; AR, arthritis; MR, malar rash; MUC, mucositis; LN, lupus nephritis; HE, hematologic disorder; IM, immunologic disorder; ANA, anti-nuclear antibody; ASA, aspirin; CYC, cyclophosphamide; HCQ, hydroxychloroquine; IVP, IV methylprednisolone; MMF, mycophenolate mofetil; MTX, methotrexate; PRED, prednisone*.

*^a^Medications patient taking at time of sample collection*.

In addition, we tested for CD4^+^ T-cell exhaustion status in the active disease state by analyzing T-cell responsiveness to SEB as well as PD-1 expression on the various CD4^+^ T-cell subsets (Teff, Tregs, and Tnaïve/mem).

## Results

### Case Presentations

#### A-cSLE Patient (New Diagnosis)

Our A-cSLE patient is a 9-year-old African-American female who was previously healthy. Three weeks prior to hospitalization, she began to experience fatigue, intermittent tactile fevers, myalgias, and arthralgias. The week prior to admission, she had daily fevers, thigh pain, sore throat, diffuse abdominal pain, decreased appetite, and a 1.5-kg weight loss. She was admitted to the general pediatrics service for further evaluation. Her physical examination was concerning for telangiectasias over the cheeks, non-ulcerative desquamation on the right buccal mucosa, multiple pin-point petechiae on the posterior hard palate, non-tender cervical and inguinal lymphadenopathy, as well as warmth and 2+ effusions over the bilateral knees, ankles, wrists, and metacarpophalangeal and proximal interphalangeal joints. Her complete blood count demonstrated a WBC of 2.06, ANC of 980, ALC of 1,200, and hemoglobin of 10.6. She also had LDH elevated to 850, ESR elevated to 83, and a positive DAT IgG. Multiple subspecialties convened to facilitate an underlying diagnosis. cSLE was considered given the cytopenias, elevated ESR, lymphadenopathy, mucosal lesions, and symmetric polyarthritis. Table 1 presents ACR classification criteria for new cSLE diagnosis for this patient. Notable immunologic markers included anti-dsDNA, anti-Scl-70, anti-SSA, and anti-cardiolipin IgG and IgM antibodies. SLEDAI at time of initial diagnosis prior to start of immunosuppressive therapy was 14, and comprised of arthritis, rash, mucosal ulcers, low complement (C3/C4), increased DNA binding, fever, and leukopenia.

Her induction regimen included 3 days of IV methylprednisolone 30 mg/kg/dose, followed by weekly IV methylprednisolone 30 mg/kg/dose for 8 weeks, daily oral prednisone 2 mg/kg/day, hydroxychloroquine 5 mg/kg/day, aspirin 81 mg/day, and weekly subcutaneous methotrexate 15 mg/mg^2^/dose. The latter was initiated as polyarthritis was a prominent feature of her initial cSLE presentation. Aspirin was included in her medication regimen to decrease risk of thrombus formation given positive antiphospholipids as outlined above. Table 2 presents key cSLE laboratories trended from initial diagnosis and throughout immunosuppressive therapies. Between time points 1 and 3 (total span of 80 days), the patient was admitted twice for fever and severe neutropenia without an identifiable source of infection. Both episodes of febrile neutropenia resolved after administration of IV methylprednisolone 30 mg/kg/dose. SLEDAI decreased over the three time points, however, SLEDAI at the third time point indicated ongoing disease activity (Table 1).

**Table 2:** **Key laboratory values found prior to, during, and after initiation of therapies in patient with active childhood-onset systemic lupus erythematous (cSLE)**.

Key laboratories (normal values)	Laboratory values for active cSLE patient		
	Initial admission (time point 1)	One month after start of therapy (time point 2)	Three months after start of therapy (time point 3)
HgB (11,500–15,500)	10,600	10,000	10,300
ALC (1,500–6,500)	1,200	1,510	3,860
C3/C4 (C3: 92–200) (C4: 12–45)	31/7	47/7	48/5

*ALC, absolute lymphocyte count; HgB, hemoglobin*.

#### Inactive cSLE Patient

Inactive cSLE patient is a 17-year-old African-American female with cSLE of 8 years duration prior to inclusion in this study. Table 1 lists the ACR classification criteria fulfilled at initial diagnosis. Immunologic markers at time of initial diagnosis included positive anti-SSA, anti-cardiolipin IgG, and anti-dsDNA antibodies. Renal biopsy performed during initial diagnosis demonstrated class IV/V lupus nephritis, necessitating six monthly pulse doses of IV cyclophosphamide 1,000 mg/m^2^/dose. Other medications used after initial cSLE diagnosis to induce remission included weekly IV methylprednisolone 30 mg/kg/dose, daily oral prednisone 2 mg/kg/day, daily hydroxychloroquine 5 mg/kg/day, and daily aspirin 81 mg/day given positive anti-cardiolipin IgG. After completion of her induction regimen with six monthly pulse doses of IV Cyclophosphamide, the patient was transitioned to daily mycophenolate mofetil 500 mg/m^2^/dose BID as a maintenance medication. Medications that this patient was taking at the time of our sample collection are listed in Table 1 with their respective superscripts. Her ACR classification criteria at the time of sample collection were positive ANA. SLEDAI at time of enrollment for this study was 0, reflecting no demonstrable features of disease activity.

#### Flow Cytometry Results

##### Frequency of Peripheral Blood CD4^+^CD25^+^Foxp3^+^ and CD25^hi^ Foxp3^hi^ Tregs

**Figure 2:**
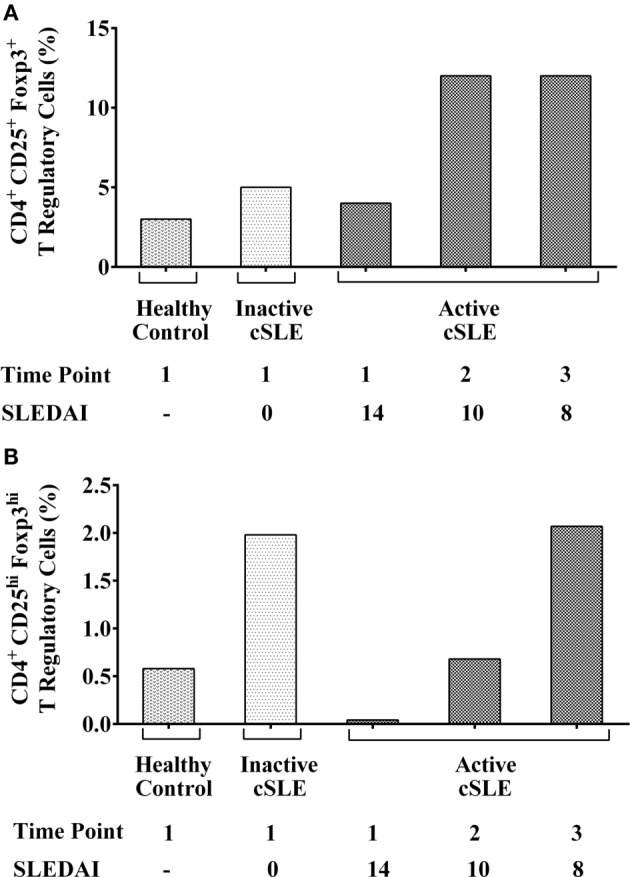
**(A)** Frequency of peripheral blood CD4^+^CD25^+^Foxp3^+^ regulatory T-cells (Tregs) in I-cSLE, active cSLE (A-cSLE) patient, and healthy control (HC). Frequency of Tregs (CD4^+^CD25^+^Foxp3^+^) under basal conditions in the HC, inactive childhood-onset systemic lupus erythematous (cSLE) patient, and A-cSLE patient. **(B)** Frequency of CD25^hi^Foxp3^hi^ peripheral blood Tregs in I-cSLE patient, A-cSLE patient, and HC. Frequencies of CD4^+^CD25^hi^ Foxp3^hi^ under basal conditions in the HC, inactive cSLE patient, and A-cSLE patient.

Both the inactive (I-cSLE) and A-cSLE patients possessed high frequencies of CD4^+^CD25^+^Foxp3^+^ Tregs (5–12%) compared to the HC, who had 3%Tregs (Figure 2A). In the A-cSLE patient, gradual improvement in disease activity from time points 1 to 3 (SLEDAI 14 → 10 → 8) correlated with an overall increase in Tregs percentages: 4 → 12 → 12% (Figure 2A). The active lupus patient also had a higher percentage of CD25^hi^Foxp3^hi^ Tregs at time points 2 and 3 (after initiation of therapy), but not at time point 1 compared to the HC (Figure 2B). In the A-cSLE patient, the percentage of CD25^hi^Foxp3^hi^ Tregs inversely correlated with their disease activity; CD25^hi^Foxp3^hi^ Tregs/SLEDAI score: 0.04%/14, 0.68%/10, and 2.07%/8. Collectively, these results suggested that both our inactive and active lupus patients were not Tregs deficient and that the percentage of CD25^hi^Foxp3^hi^ Tregs increased after therapy was initiated in the newly diagnosed patient.

##### Ratio of Tregs/Teff CD4^+^ T-Cells

**Figure 3:**
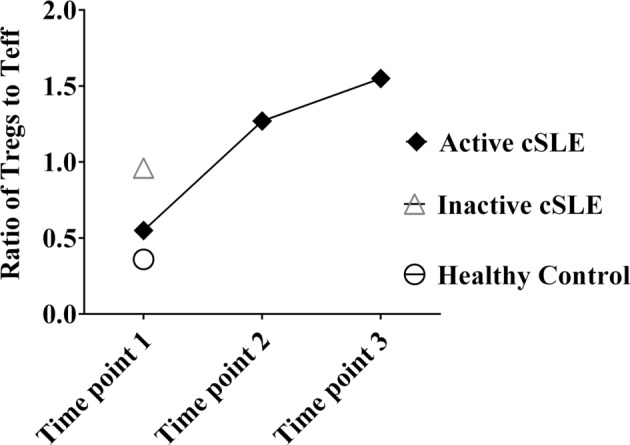
**Ratio of regulatory T-cells to Teff in active cSLE (A-cSLE) patient over time points compared to I-cSLE and healthy control (HC)**. Samples from A-cSLE patient were collected at three time points (1, 2, and 3). Samples from HC and I-cSLE patient were collected once, represented as time point 1.

Since the efficacy of Tregs suppression is shown to be dependent on Tregs/Teff ratio (9), we evaluated for differences between this ratio among the active patient, inactive patient, and HC. In the A-cSLE patient, the Tregs/Teff ratio gradually increased over time points 1, 2, and 3 from 0.55 → 1.3 → 1.6, respectively. In the HC, this ratio was 0.36, and in the I-cSLE patient, the ratio was 0.96 (Figure 3). The Tregs/Teff ratio was highest in the active patient at time point 3 and correlated with their lowest SLEDAI of 8. These observations show that active lupus can lead to an altered Tregs to Teff ratio, and that this immunologic homeostasis changes with therapy.

##### T-Cell Exhaustion Status: Responsiveness to SEB Stimulation and PD-1 Expression

**Figure 4:**
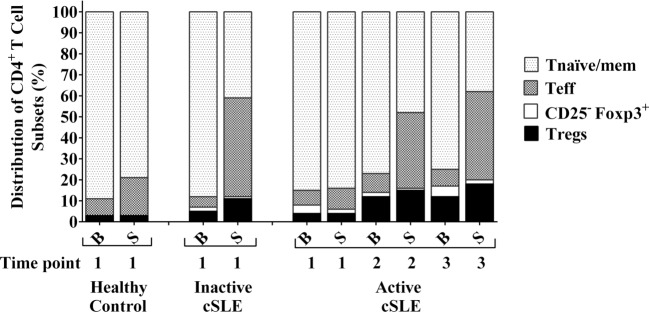
**Distribution of CD4^+^ T-cell subsets in I-cSLE patient, active cSLE patient, and healthy control under basal and post-stimulatory conditions**. Peripheral blood mononuclear cells were stimulated (S) with super-antigen staphylococcal enterotoxin B for 24 h and then compared with their corresponding samples under basal conditions (B) for frequencies of different CD4^+^ T-cell subsets.

There were no significant differences in the percentages of CD4^+^ Teff or Tnaïve/mem cells at time point 1 under basal (non-stimulated) conditions between the HC and lupus patients (Figure 4). The HC had 7.93% Teff cells, I-cSLE patient had 5.12% Teff cells, and A-cSLE patient had 6.76, 9.34, and 7.67% Teff cells at time point 1, 2, and 3, respectively. The HC had 88.8% Tnaïve/mem cells, I-cSLE patient had 87.6% Tnaïve/mem cells, and A-cSLE patient had 86, 76.5, and 75.8% Tnaïve/mem cells at time point 1, 2, and 3, respectively (Figure 4).

However, stimulation with SEB demonstrated differences in the CD4^+^ Teff as well as Tnaïve/mem populations between the HC, inactive, and active lupus patient. This is best demonstrated by the term “fold change” in CD4^+^ Teff cells upon stimulation with SEB, defined as the frequency of Teff post-stimulation divided by the frequency of Teff pre-stimulation. This value was notably highest in the I-cSLE patient compared to the HC and the A-cSLE patient with a 9.10-fold increase in the percentages of CD4^+^ Teff cells after stimulation with SEB (Figure 4). Interestingly, the fold change of CD4^+^ Teff cells in the A-cSLE patient gradually increased after induction of therapy from time points 1 to 3: 1.43-fold → 3.87-fold → 5.51 (Figure 4).

With regards to fold changes in the naïve/mem CD4^+^ T-cell compartment, defined as the frequency of Tnaïve/mem cells post-stimulation divided by the frequency of Tnaïve/mem cells pre-stimulation, there was a 1.2-fold decrease in the HC, while the I-cSLE patient showed 2-fold reduction in this subset (Figure 4). In the A-cSLE patient, this reduction in the Tnaïve/mem subset became more pronounced from time points 1 to 3 from 1.02-fold → 1.6-fold → 2.04-fold as her disease activity gradually improved with SLEDAI scores decreasing from 14 → 10 → 8 (Figure 4). In summary, we observed heightened CD4^+^ T-cell responsiveness to SEB stimulation in the inactive cSLE patient as well as the A-cSLE patient at time points 2 and 3 compared to the HC. In other words, heightened CD4^+^ T-cell responsiveness was noted with lower disease severity in our cSLE patients.

**Figure 5:**
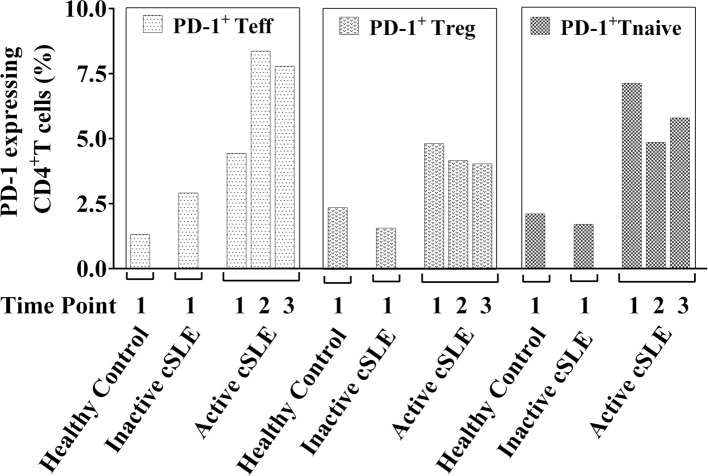
**Surface expression of PD-1 on Teff, regulatory T-cells (Tregs), and Tnaive/mem cells under basal conditions**. Frequencies of PD-1 expressing Teff, Tregs, and Tnaive/mem cells under basal (non-stimulatory) conditions in I-cSLE patient, active cSLE patient, and healthy control.

We also checked frequency of PD-1 on different CD4^+^ T-cell subsets including Teff, Tregs, and Tnaïve/mem cells to assess for differences between active and inactive lupus patients as PD-1 has been shown to play a role in T-cell exhaustion (23). PD-1 was expressed at much higher percentages on CD4^+^ Teff, Tregs, and Tnaïve/mem cells at all three time points in the A-cSLE patient compared to the I-cSLE patient and HC (Figure 5). The active patient had PD-1 expressing Teff cells that ranged from 4.4 to 8.37%, the inactive patient had 2.9% PD-1 expressing Teff cells, and the HC had 1%. Similarly, the active patient had PD-1 expressing Tregs that ranged from 4.0 to 4.81%, the inactive patient had 1.6% PD-1 expressing Tregs, and the HC had 2.35%. Regarding PD-1 expressing Tnaive/mem cells, the HC had 2.11%, the inactive patient had 1.71%, and the active patients had 5.8–7.13%.

## Discussion

In this observational study, we looked at the longitudinal changes in the distribution of CD4^+^ T-cell subsets as well as T-cell exhaustion status in a newly diagnosed pediatric lupus patient, following trends with initiation of immunosuppressive therapy, and then comparing these values to an inactive cSLE patient and a HC. The size of this study precludes any generalizable conclusions, but helps generate certain hypotheses regarding relevant immunologic endpoints in cSLE patients. For example, in the case of our active lupus patient, we observed that the intra-individual trends in immune cell composition may be more important than inter-individual differences to better understand changes in the T-cell compartment of lupus patients during immunosuppressive therapy.

Our results indicate that both the inactive and A-cSLE patients from our study were not Tregs deficient compared to age-matched normative data (24, 25). Furthermore, the percentage of CD4^+^CD25^hi^Foxp3^hi^ T-cells, a subset which has been noted to have increased suppressive ability, also fall within published reference ranges (26, 27).

One explanation for the significantly increased frequency of Tregs in our active lupus patient could be the effect of immunosuppressive therapies, such as glucocorticoids and cyclophosphamide. These medications have been shown to increase Tregs frequency in SLE as well as other conditions (28–31). In a study by Karagiannidis et al. on asthmatic patients, the use of glucocorticoids significantly increased Foxp3 mRNA expression (29). In another study on myasthenia gravis patients by Fattorossi et al., Tregs in the peripheral blood were significantly lower in untreated patients, whereas they were normal or elevated in patients on corticosteroids (30).

Another hypothesis generated from this study is that in order to balance Teff cell activity and restore immune homeostasis, the number of Tregs and the ratio of Tregs/Teff cells must increase. Perhaps a higher frequency of CD4^+^ Tregs is required as a form of functional compensation to maintain immune homeostasis over hyperactive CD4^+^ T-cells in these patients, suggesting that the Tregs/Teff ratio may be a better indicator of a patient’s clinical presentation compared to Tregs frequencies alone.

By using SEB stimulation, we noted that CD4^+^ T-cells in the inactive cSLE patient had a heightened response to the antigen, which enabled them to rapidly acquire an activated phenotype from the naive or resting status. Conversely, in the active patient, CD4^+^ T-cells were relatively hypo-responsive to SEB stimulation when disease activity was higher (time point 1). This observation suggests that CD4^+^ T-cells in the inactive cSLE patient was hyper-responsive or possessed a lower threshold of TCR activation compared to the HC and active lupus patient. Tsokos et al. have previously described similar biochemical abnormalities of SLE T-cells, noting a hyperexitable phenotype secondary to heightened calcium responses following TCR activation due to a modified T-cell receptor/CD3 complex (9, 32). Literature has also shown that isolated SLE T-cells display pre-clustered lipid rafts to aid in the formation of the immunological synapses, indicating that the T-cells are “poised” for activation (9).

The A-cSLE patient in this study had higher frequencies of PD-1^+^ expressing Teff, Tregs, and Tnaïve/mem cells at all time points compared to the HC and inactive lupus patient. Previous reports have shown that elevated levels of PD-1 on T-cells in the setting of chronic infection or antigen exposure may function to dampen T-cell function in order to control ongoing inflammation and tissue damage (33). Taken together, this would suggest that our active lupus patient possessed T-cells that were in an exhausted state. The concept of T-cell exhaustion in this patient is also plausible from active disease and inflammation secondary to chronic antigen stimulation, which would create a progressively hypoxic microenvironment, and in turn downregulation of the TCRζ chain (34–36). This would render their T-cells hypo-responsive to antigenic stimulation, as mentioned in our observation above.

In summary, in this pilot funded study, we feature illustrative and hypothesis-generating cases of pediatric lupus. As a platform for future studies, we postulate from our results that following overall intra-individual trends in immune components as well as ratios may allow for a better gauge of disease improvement after initiation of therapy. As this study is by no means conclusive, more detailed studies with larger cohorts, different ethnic groups, and additional markers, including functional cytokine profiles, are necessary to elucidate the regulatory mechanisms of Tregs as well as co-inhibitory molecules in pediatric lupus. If successful and validated in larger studies, this approach could be applied to improve immune surveillance of cSLE patients—a subset of rheumatology with a paucity of literature as it relates to immune profiling.

## Ethics Statement

Prior to sample collection, consent to publish was obtained from each patient’s parent as they were recruited under an immune profiling research protocol approved by the Institutional Review Board of Baylor College of Medicine (#H33095).

## Author Contributions

SS contributed to the design of the study, acquired data, performed data analysis and interpretation, drafted the initial manuscript, reviewed and revised the manuscript, and approved the final manuscript as submitted. SW, EM, and AG contributed to the design of the study, supervised data collection, reviewed and revised the manuscript, and approved the final manuscript as submitted. JO and GM conceptualized the study, critically reviewed the manuscript, and approved the final manuscript as submitted. All authors approved the final manuscript as submitted and agree to be accountable for all aspects of the work.

## Conflict of Interest Statement

The research was conducted in the absence of any commercial or financial relationships that could be construed as a potential conflict of interest.

## Abbreviations

cSLE, childhood-onset systemic lupus erythematosus; Tregs, regulatory T-cell; Teff, effector T-cell; Tnaïve, naïve T-cell; PD-1, programmed death-1 receptor; SEB, staphylococcal Enterotoxin B; PBMC, peripheral blood mononuclear cell; SLEDAI, systemic lupus erythematous disease activity index.
